# Supplementary material for: Pre-Exposure Prophylaxis (PrEP) Adherence Questionnaire: Psychometric Validation among Sexually Transmitted Infection Patients in China
Source: Int J Environ Res Public Health. 2021 Oct 19;18(20):10980. doi: 10.3390/ijerph182010980 (PMC8535751; doi:10.3390/ijerph182010980)
Supplement: Supplementary file 1 [file ijerph-18-10980-s001.zip › ijerph-1341516-supplementary.pdf]

PrEP Adherence Questionnaire

Would you agree with the following statements about free oral pre-exposure prophylaxis (PrEP)? For each statement, please choose the option that best fits your situation.

Item	disagree	unclear	agree
1. PrEP can effectively reduce your risk of HIV infection.			
2. This medication could reduce the risk of HIV transmission to your partner.			
3*. The side effects of PrEP can affect your daily life.			
4*. My partner would think I do not trust him/her, if they find me taking this medication.			
5. A lot of my friend would be willing to take PrEP.			
6. My partner is supportive for me to take PrEP.			
7. You are confident to use free PrEP if you want to.			
8. It is up to me whether to take PrEP if PrEP is free to access.			

PS: The questionnaire included four scales: benefit, barrier, peer support and self-

efficacy. Benefit refers to perceived benefit when one take PrEP strategy, including item 1-2; Barrier refers to perceived barrier when one take PrEP strategy, including item 3-4; Peer support refers to support get from peers when one take PrEP strategy, including item 5-6; Self-efficacy refers to one 's belief in his/her own ability to take PrEP strategy, including item 7-8.

Each item on the questionnaire was scored using a three-point scale: 0 points for "disagree", 1 point for "unclear", and 2 points for "agree"; the total score on the questionnaire was 16 points. Asterisked items should be scored in reverse. Higher scores indicated higher adherence to PrEP.
